# Recycling of Mining Waste in the Production of Masonry Units

**DOI:** 10.3390/ma15020594

**Published:** 2022-01-13

**Authors:** Nicoleta Cobîrzan, Radu Muntean, Gyorgy Thalmaier, Raluca-Andreea Felseghi

**Affiliations:** 1 Faculty of Civil Engineering, Technical University of Cluj-Napoca, Constantin Daicoviciu Street, No. 15, 400020 Cluj-Napoca, Romania; Nicoleta.Cobarzan@ccm.utcluj.ro; 2 Faculty of Civil Engineering, Transilvania University of Brașov, Bd. Eroilor, No. 29, 500036 Brașov, Romania; radu.m@unitbv.ro; 3 Faculty of Materials and Environmental Engineering, Technical University of Cluj-Napoca, Bd. Muncii, No. 103–105, 400641 Cluj-Napoca, Romania; Gyorgy.Thalmaier@sim.utcluj.ro; 4 Faculty of Electrical Engineering and Computer Science, “Ștefan cel Mare” University of Suceava, Universităţii Street, No. 13, 720229 Suceava, Romania

**Keywords:** circular economy, construction materials, greener environment, masonry, mining waste, resources conservation, value-added properties

## Abstract

Masonry units made of clay or Autoclaved Aerated Concrete (AAC) are widely used in constructions from Romania and other countries. Masonry units with superior mechanical and thermal characteristics can improve the energy efficiency of buildings, especially when they are used as the main solutions for building envelope construction. Their production in recent years has increased vertiginously to meet the increased demand. Manufactured with diversified geometries, different mechanical and/or thermal characteristics have a high volume in the mass of the building and a major influence in their carbon footprint. Starting from the current context regarding the target imposed by the long-term strategy of built environment decarbonization, the aim of the paper is to analyze the potential of reusing mining waste in the production of masonry units. Mining waste represents the highest share of waste generated at national level and may represent a valuable resource for the construction industry, facilitating the creation of new jobs and support for economic development. This review presents the interest in integrating mining wastes in masonry unit production and the technical characteristics of the masonry units in which they have been used as raw materials in different percentages. Critical assessment framework using SWOT analysis highlights the key sustainability aspects (technical, environmental, social, economic) providing a comprehensive and systematic analysis of the advantages and disadvantages regarding the integration of mining waste as secondary raw materials into masonry units production.

## 1. Introduction

Construction, building operation, and the construction industry in general contribute more than 39% of total global CO_2_ emissions [1], playing a key role in achieving the long-term goals set out in the Paris Agreement (2015) [2] to “*keep the temperature rise to 1.5 °C compared to the industrialization period*” and to ensure the transition to climate neutrality by 2050 [3].

To meet these goals, the EU’s development priorities set out in the “European Green Deal” [3] call for increasing the rate of building renovation making them more energy efficient and development of the industrial sector based on sustainable principles to support the transition from linear to circular economy [4,5]. The circular economy is a new paradigm that aims to keep materials in the economy for a very long time, thus limiting the generation of waste and exploitation of primary natural resources [6,7].

The circular economy requires recycling of materials considered waste and leads to major changes in products and systems design, re-adaptation of technological flow (supply and distribution chains), or company-specific business model [3,8]. Extension of digital passport for all products to sustain waste circularity [9] and population education about the advantages obtained by using materials made of recycled waste are also imperative in this regard.

Recycling of waste to preserve the natural resources and to reduce disposal [10] will reduce the environmental impact by contributing to the growth of circular economy above the value of 9% currently recorded [11], which without climate neutrality cannot be ensured. Promoting waste recycling to reduce excessive consumption of natural resources in the U.E. and/or dependence on imports emerges also from the Raw Materials Initiative (RMI) launched in 2008 to identify the level of needs and mineral resources for each EU country [12].

**Figure 1:**
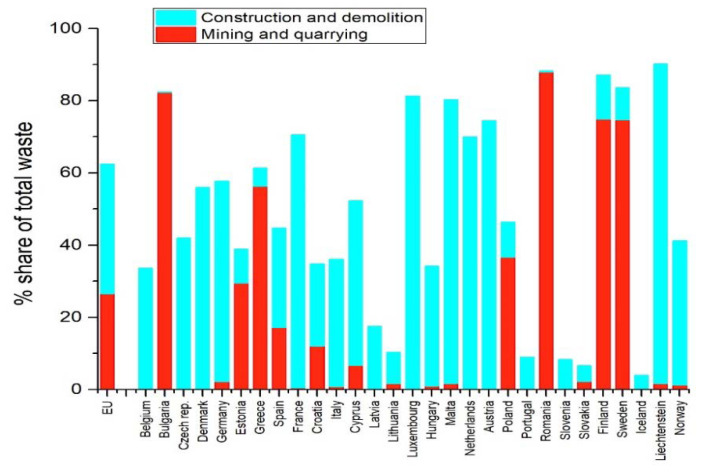
Waste generation by European countries, 2018 [16].

Romania is a country rich in non-metallic mineral resources (sand, clay minerals, bentonite, limestone, graphite, perlite, diatomite) and metalliferous (copper ore, lead, zinc, aluminum, gold, manganese chromium, nickel, tungsten) [13], whose exploitation has generated large amounts of waste over time (Figure 1). Mining of copper, manganese, zinc, gold, uranium has greatly diminished since the 1990s as a result of industrial restructuring and the closure of most of the operational mines, declared inefficient [14,15]. The strategic objectives provided by the “Mining Industry Strategy developed for the period 2017–2035” aim to increase the use of domestic mining resources to reduce dependence on imports of mineral raw materials, which are vital for the development and sustainable growth of the Romanian economy [15], but also for development of mining areas that have now become disadvantaged areas being characterized by a low living standard and lack of jobs. It is necessary to use innovative technologies of “mineral resources” starting from cradle to exploitation, processing, or recovery of waste.

The mining industry, during its existence has significantly affected the environment, a supplementary reason for which the recovery of mining waste in various areas are stringent issues raised nowadays. An important direction in this regard is to find new techniques and technologies for the recovery of waste, exploiting this valuable resource in terms of economic growth [15].

According to statistical data valid for 2018 and published by Eurostat [16], the quantity of waste generated in the European Union by all economic activities and households was over 2300 million tons. As a percentage, the waste from construction and demolition and those from mining and quarrying activities represent the largest share, 35.9% and 26.6%, respectively (Figure 1).

Much waste from mining and construction activities are classified as major mineral wastes, being different from other types of wastes, and represent about three quarters (74% or 5.2 tons per capita) of total waste generated in 2018 at the EU level. The higher levels of major mineral waste recorded in EU countries are recorded in areas with important mining and quarrying activities, such as Romania, Finland, Sweden, or Bulgaria.

According to Figure 1, Romania generates approximately 88% of mining waste from exploitation and processing activities of mineral resources (mining, quarrying activities) and only 0.3% from construction activities (from all data recorded), out of a total of 203 million tons of waste.

**Figure 2:**
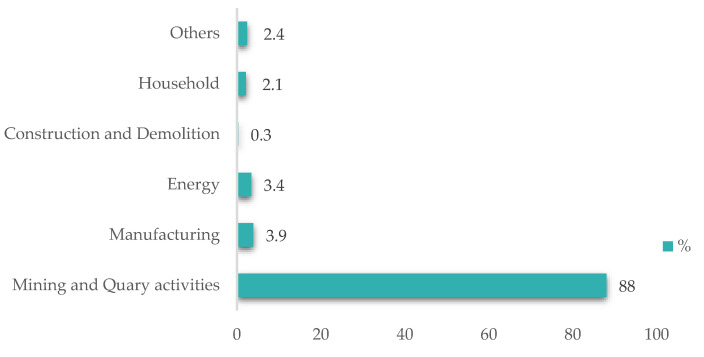
Percent of waste by types of activities in Romania, 2018 [16].

The distribution of waste generated by various economic and household activities at the national level is presented in Figure 2.

In concrete figures, in 2018, a quantity of 178.6 million tons of mining waste was generated, increasing compared to the previous years, for which, only 8.9 million tons were recovered in various forms [17].

The use of mining waste in construction may play a significant role for the conservation of natural resources, but also for reducing the impact on the environment or human health due to inadequate storage.

Extractive industrial waste was defined and classified initially by Directive 75/442/EEC [18] and later by Directive 2014/955/EU [19] in four major groups: tailings; waste rock; sewage sludge; topsoil.

Tailings are solid wastes or sludges that remain after mineral treatment by separation processes (e.g., grinding, crushing, sizing, flotation, and other physico-chemical techniques) to extract valuable minerals from a lesser rock, while waste rocks are economically unprofitable rocks that are excavated to gain access to ore. Sewage sludge results during exploitation by sedimentation in flooded quarries or tailings ponds and may contain large amounts of heavy metals, non-metals (arsenic), and neutral salts (sulfate, calcium). The vegetal soil is the upper layer of the land surface (after removing the roots and capitalizing of wood) and the lower layer of soil up to the rock layer; although identified as waste, these soil materials can be stored in stacks and reused for vegetation resettlement. Hazardous mining waste can contain harmful substances and toxic metals [20], which can cause environmental disasters and endanger human health or habitat destruction. In contrast, in the case of non-hazardous and inert mining waste, the quantities of heavy metals are reduced and may represent a valuable resource for the production of new materials [20] after pre-treatment.

By analyzing the current literature, where information is dissipated in various sources, there is a critical necessity to investigate, compile, and elect the main information reachable to assist further development of mining waste circularity. Therefore, this review is intended to emphasize comprehensive information on mining waste used in the construction materials sector, specifically for masonry units’ production, and identifies the key sustainability aspects from technical, economic, social, and environmental points of view.

The major contributions of this review are:

- comprehensive review on integrating mining waste used for masonry unit’s production (clay masonry units, autoclaved aerated concrete units, and geopolymers masonry units);
- using the “SWOT” model to conduct a critical evaluation framework;
- overall, this review article may represent a preliminary study for academics, scientific researchers, and industry to further identify the feasibility parameters and limitations for exploiting mining waste as secondary raw materials in the production of masonry units.

Further, the main highlights of this work can be summarized as follows:

- reasons for recycling mining waste;
- overview of the masonry units on the transition to the circular economy context in the brick industry to produce eco-responsible construction materials;
- discussions regarding the influence of the mining waste chemical and mineralogical composition on the manufacturing technological process of new construction materials;
- discussed the effect of incorporating mining waste into masonry unit’s production (clay masonry units, autoclaved aerated concrete units, and polymers masonry units);
- over 100 scientific papers were reviewed to present an overview of masonry developments over the time and to the discuss the role of mining waste as a raw material in the production of masonry units.

The structure of this paper is described as follows. First, a general overview on masonry units in transition to the circular economy is addressed, making assessments of clay masonry, autoclaved aerated concrete, and geopolymers masonry units, but also on chemical composition and mineralogical composition of mining waste. Afterward, the various approaches in the literature related to mining waste for clay masonry units’ production, mining waste for autoclaved aerated concrete production, and mining waste for geopolymer masonry units’ production were presented and discussed. Following these discussions, a critical evaluation framework was developed that uses SWOT analysis to identify the advantages and pitfalls of the integration of mining waste as secondary raw materials into masonry units’ body. Finally, conclusions were drawn that summarize the main appraisals on mining waste as secondary raw materials used for the production of masonry units based on the current discussion. This review provides a good basis and guidelines for encouraging the wide adoption of mining waste circularity in masonry unit production for sustainable construction.

## 2. Masonry Units: An Overview

Masonry units (clay brick and AAC) are used for the construction of structural or non-structural walls based on their mechanical properties. The group of masonry unit, the wall density, volume of cavities (%), seismic area, and mechanical characteristics are defining factors in selecting the height regime of masonry buildings. These criteria are regulated by the Romanian codes P100-1/2013 [21], CR6-2013 [22], and must be analyzed in the preliminary stage of the building design.

In the case of framed structures, the walls being infilled can be constructed with masonry units with lower compressive strength, if adequate constructive measures are provided to prevent their failure during a strong earthquake.

The restrictions given by design codes limit the use of masonry units with lower compressive strength than 5 MPa to the construction of masonry buildings placed in seismic areas, which are specific to Romanian territory.

The content of soluble salts and the highwater absorption of units can affect the durability of masonry work and their lifespan, making necessary the rehabilitation work that has the effect the increasing embodied energy of building.

The transition to climate neutrality involves a drastic reduction of CO_2_ emissions in construction sector, especially in buildings, so that the uses of materials with improved thermal characteristics or/and low embodied energy for construction of envelope members are required and have become a necessity.

The development of building materials with high mechanical strength and/or thermal properties and/or low embodied energy has now become a major challenge for masonry unit manufacturing. Replacing conventional with secondary raw materials resulted from industrial and mining activities can be an effective solution in ensuring climate neutrality targets.

### 2.1. Clay Masonry Unit

Fired bricks are materials with wide applications in the construction sector, influencing energy consumption in buildings, greenhouse gasses emissions, but also the costs related to heating and cooling the buildings during operation. Fired at temperatures up to 900 °C required for mineralogical transformation of raw materials and for brick sintering, they have a major impact on the environment.

It is imperative in the transition towards a circular economy in the brick industry to produce materials with high thermal characteristics and/or low embodied energy, long lifespan, and possibilities for reusing or recycling at the end of lifecycle so as to keep the product in economy as long as possible. Clay bricks have a long lifecycle up to 150 years [23,24] and can be reused after building demolition for new masonry works if their physical and mechanical characteristics are adequate. Recycling the secondary raw materials in the brick and other industries may also be a viable strategy for “closing the loop”.

Physical, mechanical, and thermal characteristics of clay units are in accordance with chemical composition or mineralogical content of raw materials (primary or secondary), materials granulometry, firing temperature, porosity, and forming technology (pressure, extrusion, geo-polymerization) [25]. Based on targeted characteristics, the composition of the clay matrix can be optimized so as to meet the required criteria in terms of sustainability and the circular economy.

In recent decades, the brick industry has evolved continuously, producing a wide range of masonry units (solid and perforated bricks, blocks with vertical or horizontal cavities) [23,24], whose thermal characteristics have been improved to meet the thermal requirements imposed by norms. As can be seen in the scientific literature, the thermal properties of bricks was optimized by increasing the volume of cavities, by changing the cavity geometry or the profile of exterior surface [26,27,28,29], and by filling the cavities with different thermal insulation materials [30,31,32,33,34]. Another solution studied and adopted for ceramic product optimization in terms of embodied energy or thermal performance was the incorporation in the clay matrix, organic or inorganic waste [35,36], resulted from different activities.

Organic waste (agricultural, food, industry waste, etc.) incorporated in different percentages in clay body as pore forming and/or fluxing have been investigated by different authors [36,37,38,39] highlighting economic, mechanical, or thermal advantages, respectively, and the caloric value [40] to contribute to circular economy.

Inorganic waste with role of degreaser and/or fluxing being used in the clay matrix to reduce plasticity [41,42] and shrinkage to drying or firing or to increase porosity have a lower impact on the environment, but require higher water consumption. Construction and demolition waste [43,44], cutting and processing of natural stone [45,46,47], mining activities [20,41,48,49,50,51,52,53,54,55,56,57,58,59,60,61,62,63], or industrial waste are an alternative solution with high potential in the manufacturing of clay units.

Many of these measures are aimed at increasing thermal performance and/or reducing the embodied energy of the products. The limitations given by the geometric features and characteristics, especially when are used in seismic areas or by the mechanical strength have been intensively analyzed in the literature. Some of the identified solutions can be implemented at the producer level, but not all of them are efficient and economic. Finding feasible solutions with wide applicability in the field is becoming more stringent to reduce the environmental impact and to preserve the primary resources.

### 2.2. AAC Masonry Units

AAC units are produced by autoclaving and have a high porosity of up to 50–60% from mass. They are usually made of cement, lime, silicious materials, gypsum, and aluminum powder [64] as pore forming agent [65].

Depending on the mechanical and geometrical characteristics, AAC units can be used to construct the structural wall in case of masonry buildings with lower height regime or to construct non-structural wall (for infill and partitioning). They have good thermal characteristics and low density, which can contribute in the reduction of the building mass and the related seismic force. ACC bricks are considered eco-friendly materials [66] due to the lower embodied energy compared to that of fired masonry units.

The use of waste in the production of AAC units has been analyzed by various researchers to reduce CO_2_ emissions and to conserve natural resources. They have been used as substitutes for silicious materials, cement, or aluminum [67] in various percentages, so that their mechanical properties to remain within the acceptable limits provided by standards or design codes.

The potential of using waste in autoclaved aerated concrete has been studied by [68], coal bottom ash by [69], silica fume and fly ash by [70], industrial and agricultural waste by [71], perlite waste by [72], rice husk ash by [73], graphite tailings by [74], copper tailings by [75,76], hematite tailings by [77], iron tailings by [78,79,80,81], silicon tailings by [82], coal gangue by [83], etc. Studies have shown the feasibility of using secondary raw materials as a substitute for virgin materials.

The mechanical characteristics of AAC units are influenced by the materials’ chemical and mineralogical content, but also by the pore distribution and the porosity of the final product.

The formation of crystalline phases (Tobermorite—Ca_5_Si_6_O_16_(OH)_2_·4H_2_O) is favored in the autoclaving process. The increase of soluble SiO_2_ favors the reaction with Ca(OH)_2_, thus contributing to the appearance of Calcium-Silicate-Hidrate (C-S-H) gel. Dual-alkali silicates reacts with SiO_2_ to form tobermorite with a role in increasing the material mechanical parameters and formation of C-S-H gel [66,79].

Thermal characteristics are influenced not only by the type or pore distribution, but also by the chemical content of raw materials. Low specific weight materials can significantly contribute to the reduction of thermal conductivity [66]. Also, the raw materials that favor the appearance of phase composition (crystalline or amorphous) have a favorable effect in increasing the thermal performance of materials.

### 2.3. Geopolymers Masonry Units

An alternative solution to fired clay bricks is represented by geopolymer masonry units. Geopolymers, invented and developed by Joseph Davidovits since the 1970s [84], are now also used for the production of ecological concrete or masonry units [85].

Geopolymers are obtained following a chemical reaction between aluminosilicates oxides and hydroxide and/or alkaline silicate solutions (NaOH; KOH) [85,86], processed at a temperature below 100 °C to reduce water evaporation [87]. The advantages of using geopolymers consist in saving energy and CO_2_ emissions [86].

The mechanical characteristics of the product are mainly influenced by the used raw materials, the calcination temperature, the curing time, or the used additives [88,89].

Geopolymerized bricks made of different waste have been intensively studied in recent years, such as industrial aggregates [90], fly ash, construction and demolition waste [89], tailings [91,92,93,94,95,96], etc.

## 3. Mining Waste Used for Masonry Unit’s Production

**Figure 3:**
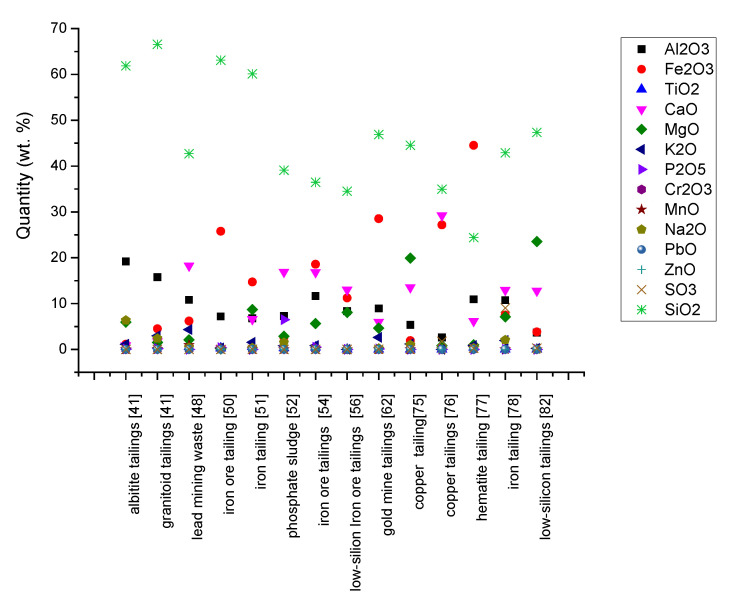
Chemical composition of waste.

**Figure 4:**
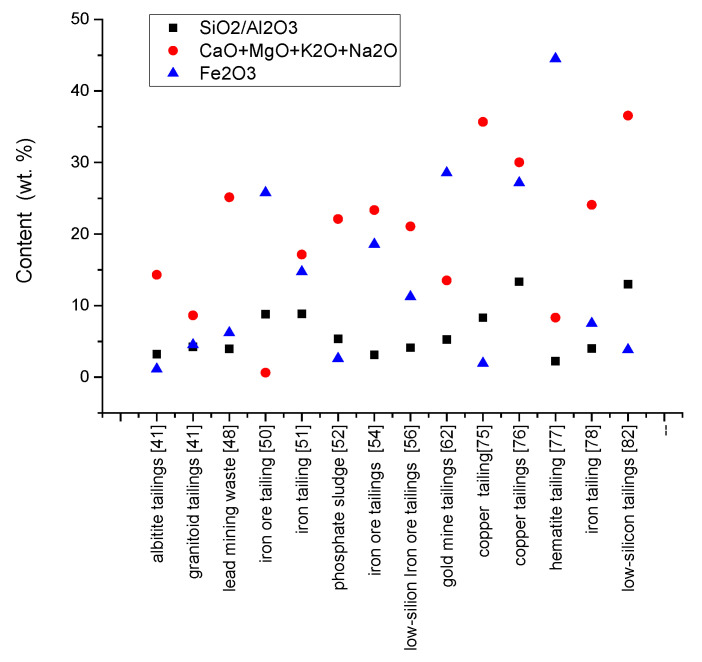
Ratio of SiO_2_/Al_2_O_3_, Fe_2_O_3_ and fluxing materials.

Knowledge about chemical content and mineralogical composition are essentially in the selection stage of secondary raw materials. They must be similar and compatible in regards to chemical and mineralogical composition with conventional ones if used as substitutes or additive in the production of new buildings materials [97] with conventional technology. Re-adaptation of technological flow to new supply chains involves technological changes and investment cost. The chemical and mineralogical content of mining waste investigated by the authors [41,48,50,51,52,54,56,62,75,76,77,78,82,83] is shown in Table 1, Figure 3 and Figure 4.

### 3.1. Chemical Composition of Mining Waste

In order to produce geopolymeric materials, the SiO_2_ and Al_2_O_3_ content of the raw materials used must be over 70% [98].

Clay used in manufactured of bricks usually contains SiO_2_ (50–60%), Al_2_O_3_ (10–20%), CaO, Fe_2_O_3_, and other oxides in low quantities [99]. The presence of quartz in the clay mass might cause the appearance of cracks during cooling, while alumina ensures resistance and quality to the final product after the mullitization process. The presence of Fe_2_O_3_ in the brick mass gives the desired color (from red to dark brown), but a high content, usually over than 9% [100], can lead to the appearance of the “black core”, when the oxygen rate is insufficient during firing. The formation of mullite (above 950 °C) causes the color of the product to become yellow or white [101] due to the Fe_2_O_3_ reaction and consumption during the mullitization process.

Also, the high content of soluble salt can favor the appearance of efflorescence, with a negative impact on durability of masonry units and works. Mining waste has different chemical composition, depending on their nature (Table 1 and Figure 3). Iron tailings have a high content of SiO_2_ and Fe_2_O_3_ and a lower content of Al_2_O_3_ and CaO [50,51]. In contrast, the granitoid and albitite tailings [41] are rich in SiO_2_ and Al_2_O_3_ and low in Fe_2_O_3_ and CaO. The SiO_2_/Al_2_O_3_ ratio represents the amount of free silica contained in the material [50]. As can be observed from Figure 3, for mining waste, the SiO_2_/Al_2_O_3_ ratio is between 2 and 13 (Figure 4) well above the kaolinite [100]. The Fe_2_O_3_ content is in the range of 1 ÷ 44% and the content of CaO + MgO + K_2_O + Na_2_O varies from 0.6 ÷ 36%.

In the case of the clays, the CaO content classifies the materials in the category of calcareous materials. A content over 6% of CaO indicates a calcareous material [101], while a content of K_2_O, Fe_2_O_3_, CaO, MgO, and TiO_2_ over 9% indicates low refractive materials.

The compositional quality of secondary raw materials may be different or lower than primary ones [102]. They can contain various pollutant soluble salts that arise the necessity to use different treatments or to use additional additives to improve the quality [44] prior to the production of the new materials.

Raw materials must be classified according to the standards and be followed by declaration of performance [97].

### 3.2. Mineralogical Composition of Mining Waste

The mineralogical composition of mining waste (Table 1) from raw materials influences the energy consumption during the manufacturing, but also the porosity and durability of products. Raw materials rich in calcite require higher firing temperature above 1100 °C [103] to complete the phase transformation of minerals. At a temperature of about 800 °C, calcite is transformed into calcium oxide, increasing the brick porosity [103,104] and decreasing the thermal conductivities and density. Great attention must be paid to the size of clay granules responsible for the transformation of calcium oxides into calcium hydroxide, a consequence of which is the volume increase and mechanical strength decrease.

Another important parameter in selecting the clay brick units is the color. The presence of iron oxide in clay matrix is essential in obtaining the reddish color. Other parameters that influence the brick color are temperature and firing time [105].

Studies carried out in recent years highlighted the potential of using mining waste in the production of all types of masonry units (Table 2, Table 3 and Table 4). Depending on the chemical and mineralogical characteristics, secondary raw materials replaced partially or totally the conventional raw materials (clay, sand, cement, etc.).

### 3.3. Mining Waste for Clay Masonry Units

The authors [41,48,49,50,53,57,59,60,61,62] studied the mining waste in the mixture of clay (Table 2), highlighting their microstructural and technical characteristics.

The authors Marrocchino et al. [41] used waste from mining (granitoid, albitite tailings) and from construction activities as secondary raw materials for brick manufacturing. The addition of 20% waste acts as degreaser and consequently contributes to the reduction of plasticity index (PI) to 22% in the case of granitoid tailings, respectively, to 20% in case of albitite tailings, reaching the optimum extrusion interval. Drying and firing shrinkage was also reduced, which can have a beneficial effect in reducing costs and the risk of cracking.

Iron tailings were analyzed by the authors [50,51,54,55,56,57,58,59] as a partial or total substitute of clay and sand in the production of bricks and ceramic materials. The chemical content of mining waste is close to the chemical content of ceramic materials and consequently can be an alternative solution to conventional raw materials [81]. The bricks were formed by uniaxial pressing or extrusion at 20–70 MPa and fired at temperatures between 850–1200 °C.

Studies conducted by Mendes et al. [50], showed that iron ore tailings (IOT) can be used in the clay matrix with a degreaser role, improving the extrusion parameters and thus contributing to the reduction of the cracks resulted during drying and firing. The optimal percentage of IOT in the brick mass was 29.1%, determined from experimental results on ceramic materials and statistical data processing. The average compressive strength of small-scaled perforated bricks made from the optimized mixture was 4.3 MPa with a water absorption of 20.9%. In terms of environmental performance, IOT based bricks can be included in the category of inert materials.

Li et al. [51] used the iron tailings (100%) to produce porous bricks, with improved thermal performance compared to dense materials. The low value of thermal conductivity of 0.032 W/(m·K), measured in the case of bricks with a porosity of 89%, highlights the potential of using the bricks in energy efficient buildings.

Yang et al. [54] investigated the low-silicon IOT bricks with fly ash in different percentage, showing the possibility of using the two recycled materials in the brick-making industry. The mechanical strengths of the samples decreased with the increase of the fly ash content, being above the minimum value required by standards (10 MPa).

Vilela et al. [55] analyzed the possibility of partially substituting the soil from the cement soil bricks with up to 40% iron ore mining waste. The density of the samples at 28 days varied between 1670 and 1750 (kg·m^−3^), with a figure 5% higher in the case of mixture with 40% addition of mining waste compared with reference. An insignificant decrease in compressive strength from 2.79 MPa (0% mining waste) to 2.66 MPa (40% mining waste) was recorded in the case of samples with an addition of 40%. The thermal conductivity increases from 1.42 W/m·K in the case of samples without mining waste to 1.59 W/m·K, due to the filling of the pore with mining waste.

Weishi et al. [56] studied low-silicon IOT bricks with addition of curing agent, obtaining at 28 days a compressive strength of up to 32 MPa, depending on initial curing temperature and the content of the stearic acid emulsion.

da Silva et al. [57] used 5% of iron ore tailings in the ceramic mass, obtaining increases in the value of flexural strength from 6.5 to 6.7 MPa, compared with the samples without additive whose flexural strength was 5.1 MPa. The porosity increases from 25.6% to 42.5% and water absorption decreased from 31% to 25.5% compared with clay samples. After firing, the color of the samples with the addition of mining waste was reddish, close to that of traditional ceramics, unlike the samples without mining waste, whose color was lighter (reddish-orange). Waste requires low or no grinding, thus contributing positively to production costs and environmental impact.

Luo et al. [58] investigated the sintered bricks made of mining waste. The optimal ratio of raw materials in the brick mass was selected based on the technical criteria as 54:30:10:6 (iron ore tailings:coal gangue:shale:sludge). It was found that material density decreases from 1740 to 1638 kg/m^3^ with the variation of the sludge ratio and the water absorption increased from 14.2% to 17.5%, being below 18%, which is the maximum accepted value. The compressive strength decreased from 18.2 to 14.2 MPa. Heavy metals (Cu, Zn, Cr, Pb) in the leachates were immobilized in the clay matrix during sintering, resulting in a leaching content in sintered brick lower than in the leaching of raw materials. The leaching efficiency was high, thus fulfilling the requirements provided in national standards.

Wang et al. [59] studied the bricks with optimal mass ratio 60:30:10 (IOT:flyash:kaolin). Based on XRD analysis, they studied the effect of heavy metals in bricks, highlighting the formation of new crystalline structure (spinel, silicate, aluminosilicate), having the effect of “fixing heavy metal” in the system and decreasing the amount of leaching (Cu, Zn, Cd, Pb). The leaching content decreases with increasing the sintering temperature. The result indicated that Zinc was immobilized at 850 °C, while Pd and Cd required increasing the temperature over 1000 °C.

The gold mine tailings rich in SiO_2_ and Fe_2_O_3_ were investigated by Wei et al. [62] to produce clay bricks. The results showed that mechanical properties increased with the increases of clay content and technological conditions (temperature and firing). At a temperature of 1000 °C, the mechanical strength of the sample with the addition of 40% clay was about 21 MPa and the water absorption decrease below the limit value accepted by the standard.

Gold tailing have also been studied by Yanggang et al. [61] in fired bricks composition. The amount of waste range between 60–90% of the clay mass, depending on the material fineness. Values over 10 MPa were obtain in the case of bricks with 60–90% fine tailings (less than 0.6 mm) and 10–25% medium tailings.

Suárez-Macías et al. [48] analyzed the possibility of introducing the tailings from a lead mine in the clay matrix. An increase of open porosity was observed with the increase of the tailing percent or the absorption of water and the decrease of compressive strength. Water absorption increased from 10.61 (0% tailing) to 29.52% (100% tailing) and the density decreased from 2010 to 1510 kg/m^3^. Compressive strength decreased from 118 to 7.2 MPa. The color of the brick varied from red to brown depending on the chemical composition and sintering temperature. The leachate from the metallic elements (Fe, Pb, Mn, and Ti) was substantially reduced with the incorporation into the clay mass, but Al, Mg, Br, Cu were not completely retained, which may require higher firing temperatures.

Loutou et al. [49], studied the possibility of valorization mining waste (residual rocks) resulted from the exploitation of phosphate ores in the production of fired clay bricks.

Phosphate sludge has been investigated by Ettoumi et al. [52] as a potential substitute of clays in the production of ceramic bricks, in proportion of up to 100%. Flexural (bending) strength was up to 13.4 MPa with a water absorption of about 12.5 for the samples fired at a temperature of 1100 °C. Increasing the firing temperature to 1100 °C was necessary to reduce the leachate content of (Na, K), respectively, to 1100 °C to reduce the content of Cr (from leachate) below the limits provided by the standard.

Bayoussef et al. [53] analyzed the micro and macrostructural characteristics of brick made of red clays resulted from phosphate mining activities and fly ash. The compressive strength of the bricks increased from 77 to 98 MPa in the case of samples with 10% admixture of fly ash, recording decreases of up to 73 and 57 MPa for samples with an addition of 20% and 30% fly ash, respectively. This is due to the growth of micropores and the appearance of microfissures in the materials mass. The density of the samples increased from 1840 to 1970 kg/m^3^ in the case of the sample with 30% addition of fly ash.

Zhu et al. [63] investigated the impermeable bricks made of tailing and gangue as mining waste from a feldspar mine in China, obtaining compressive strength of about 40 MPa in the case of specimens with addition of 60 wt% of gangue and 40 wt% of tailings. The values of compressive strength were reduced up to 5 MPa (90 wt%) with the increase of gangue content. Following the micro and macrostructural investigations performed on the permeable bricks, the optimal technological parameters were established, which lead to results above the values provided by standards. The optimum percent of residual mining waste was 80–90% (tailings 20% and 60–70% gangue) with addition of 10–20% waste ceramics to optimize the technological parameters (permeability and compressive strength). The increase of the sintering temperature led to the increase of the liquid phase generated by tailings and to the densification of the bricks with the effect of reducing their permeability.

### 3.4. Mining Waste for AAC Units

The potential of using mining waste in the production of AAC (75–80, 82, 83) has been analyzed by scientific researchers (Table 3). The complexity of the investigations performed revealed a series of advantages, but also disadvantages of using mining waste in the production of AAC. Chemical, mineralogical, and/or particle size distribution of raw materials were analyzed in the preliminary stage, then, based on preliminary results, the optimal percentage of mining waste, which can substitute the conventional raw materials established.

The macroscopical (mechanical strength, physical characteristics) and microscopical characterization (chemical content, mineralogical composition, microstructural, and thermal analysis) on the final products showed that the iron, copper, and hematite tailings can replace conventional raw materials in different percentage from products volume/weight having the mechanical characteristics in the acceptable limits required by standards.

The authors [78,80] found that the amount of C-S-H and the compressive strength of products decreases with the increase of the IOT content and their particle size in the AAC blocks’ composition. Mechanical properties of AAC investigated by Ma et al. [78] vary between 1.65 and 3.15 MPa, depending on the IOT content and silicon and tailings fineness. The increase of quartz finesse has a positive effect in the formation of hydration products (C-S-H and tobermorite), but leads to increases in energy demand for manufacturing. A recommended ratio for investigated AAC was 8:21–27:62–68:40:60:3:0.14% (cement:quiklime:siliceous:iron tailings:gypsum:Al powder), in which the compressive strength was about 2.5 MPa.

Mining waste such “coal gangue” and iron tailings have also been studied as secondary raw materials in the production of AAC. Studies have shown that the optimal percentage of mining waste in AAC mass is 40% iron tailings and 20% coal gangue [83], obtaining a compressive strength of 3.7 MPa.

Liang et al. [79] used IOT in a percentage of 30–55% in the production of AAC, with values of density and compressive strength influenced by percentage of mining waste from 604 to 590 kg/m^3^, respectively from 4.17 to 3.2 MPa. A compressive strength of about 4.17 MPa was recorded in the case of samples with 40% IOT from mass.

Zhao et al. [77] used hematite tailings to produce autoclaved bricks. The optimal ratio of hematite tailings:lime:sand is 70:15:15 [%] of the mixture mass, obtaining the values of compressive strength of 21.2 MPa.

The authors Huang et al. [75] analyzed the AAC with 30% copper tailings as a lime substitute, obtaining compressive strength of 4 MPa for a density of 610 kg/m^3^. The replacement of lime with skarn-type copper tailing (SCT) contributes to savings of CO_2_ emissions that would result from calcination.

Fang et al. [76] have analyzed the autoclaved sand lime bricks with copper tailing admixture in percentages from 40% to 88% from product mass. The results show that compressive strength of samples was reduced from 24.3 to 4.6 MPa in the case of specimens with 88% of tailings compared with reference. For specimens with 50% copper tailings, the values of compressive strength were increased from 10.2 up to 20.3 MPa due to increase of lime/sand ratio.

Zhao et al. [82] have studied bricks made of low silicon tailings in 85% and cementing materials 15% from their mass. The results shows that mechanical characteristics of specimens were about 16 MPa for compressive strength and 3.8 MPa for the bending.

### 3.5. Mining Waste for Geopolymer Masonry Units

As in the previous cases, different mining wastes were utilized in manufacturing geopolymer masonry units, as presented in Table 4. Copper mine tailings have been analyzed by authors Ahmari and Zhang [92,95] as an alternative to the production at low temperature of eco-friendly bricks through geopolymerization technology. It has been shown that the compressive strength of bricks was dependent on technological conditions (forming, pressing, curing temperature), but also on the NaOH concentration. The heavy metals have been immobilized in the network of geopolymers.

The authors Beulah et al. [93] studied the geopolymer bricks made of IOT and red bricks with admixture of ground granulated blast furnace slag (GGBS). The optimum mix of bricks consisted of 70% of red clay/IOT and 30% GGBS. The values of compressive strength were 28.23 MPa (70% IOT + 30% GGBS) and 16.67 MPa (70% red clay + 30% GGBS), respectively, at 28 days. The density was 1080 kg/m^3^ and only 888 kg/m^3^ in case of samples with IOT, respectively, for red clay.

The authors Zhang et al. [94] in their research investigated the influence of NaOH molarity in geopolymerization degree and their failure process under monotonic and cyclic loading.

## 4. Critical Assessment Discussions

As can be observed in Table 2, Table 3 and Table 4, mining waste has been investigated by many researchers, showing the potential in using as secondary raw materials for the production of masonry units by substituting totally or partially the natural raw materials (clays, cement, aggregates). Investigations have been performed on waste collected from different countries, especially in areas with mining deposits and/or are scarce in natural resources.

The feasibility of the integration of mining waste as a raw material in manufacturing of masonry units is gained from the SWOT analysis based on the four key aspects (technical, environmental, social, economic) to develop the critical assessment framework (Table 5).

## 5. Conclusions

The main issues defined by the Ellen MacArthur Foundation [4] and targeted in the circular economy are to preserve the quality and economic value of products for as long as possible [109]. Masonry units can contribute substantially to the energy efficiency of buildings regardless of their destination, climatic or seismic zoning. The reduction of embodied energy, of exploitation or of the recurrent or post-use energy is necessary in order to ensure the transition from the linear to the circular economy, but also in order to achieve the long-term objectives [3,110].

The studies carried out by different authors presented and analyzed in the present synthesis highlight the feasibility of using mining waste in the production of clay and geopolymer bricks or ACC units due to their characteristics and good mechanical properties.

The use of residual waste in the production of masonry units requires the development of standards [111] based on which to establish for secondary raw materials the maximum permissible concentrations of heavy metals, soluble salt content, chemical composition, particle size, and water absorption. The optimization of the maximum percentage of waste in the mass of the material must be correlated with chemical and mineralogical characteristics of the raw materials used. The results performed at the laboratory level on small samples must be validation in the pilot factories where the products will be tested on a real scale, before their production on a large-scale. Significant variations in mechanical properties may occur for bricks/block with different geometrical features or with cavities.

The construction materials industry should focus on re-adaptation of the technological flow so that it can respond to new trends. Some advanced and innovative technological solutions are required to contribute to the reduction of pollution and CO_2_ emissions.

To increase the attractiveness of the use of secondary raw materials in the production of new ones, they must be competitive with natural resources [107,112] both economically and qualitatively. The costs associated with the recycling process (sorting, crushing, grinding, transport) must be low enough so that the cost of materials with addition of mining waste to not exceed the cost of conventional materials.

Creating government policies, supported by financial facilities to stimulate the producers of masonry units to invest in modernizing the existing technological flow, but also creating an industrial symbiosis so that the waste from mining activities is redirected in real time to construction industry or other complementary sectors. Integrating the transition process to eliminate the costs of waste storage and to reduce the impact on the environment.

In Romania, there are large deposits of mining waste (Figure 1 and Figure 2) with high potential for recovery in the construction industry. An industrial symbiosis between the mining and construction industry are imperative to reduce the environmental impact and to optimize the material properties and technological flows.

## 6. Recommendations for Further Research

Quantifying the main findings of this review investigation, the recommendation for future research directions is to address topics in dedicated works, are as follows:

- necessity for additional examinations, research, and comparative analysis having as objective the concrete effect determination of the integration of mining waste in different proportions in the masonry units, in terms of resistance to aggressive environment, non-destructive testing, microstructural studies, etc.;
- Life cycle assessment and life-cycle cost analysis of production processes that integrate mining waste into masonry units vs. classical production technologies of masonry units;
- assessment of average energy consumption and transport cost needed for using different types of mining wastes for masonry units;
- large-scale validation on samples of real dimensions and in technological production conditions similar to those existing at the manufacturer, in order to ensure the research results transfer to the economic operator;
- elaboration of materials containing technical data to inform end users about the advantages of using waste-based masonry units;
- conducting surveys (social analysis) at the level of economic and administrative agents, communities in mining areas on the feasibility, opportunity and sustainability of recycling and environmentally responsible use of mining waste to create new value chains in the construction materials industry.

Thus, more focused research is absolutely necessary to be developed in this area, and mining waste as secondary raw materials seems to be a promising contribution towards the sustainability of the construction materials industry.

## Figures and Tables

**Table 1:** Mineralogical composition of selected mining waste.

Authors	Mine Waste	Mineralogical Content	Ref.
Ettoumi et al., 2021	Phosphate sludge	calcite, dolomite, bassanite, heulandite, vermiculite, quartz, hematite, fluorapatite	[52]
Yang et al., 2014	Iron tailings	quartz, calcite, hematite, clinochlore, pyrite, amphibole	[54]
Wei et al., 2021	Gold tailings	quartz, sanidine, mica, pyrite, montmorillonite	[62]
Fang et al., 2011	Copper tailings	andradite, quartz	[76]
Zhao et al., 2012	Hematite tailings	hematite, quartz, chlorite, calcite	[77]
Ma et al., 2016	Iron tailings	gypsum quartz, albite, muscovite, calcite, terranovaite	[78]
Wang et al., 2016	Coal gangue	quartz, siderite, illite, kaolinite, montmoriolite, anorthite, muscovite	[83]

**Table 2:** Types of mining waste used in bricks.

Authors	Material	Composition	Technology/ Firing/Curing Temperature	Origin of Waste	Ref.
Marrocchino et al., 2021	bricks	plastic clay B (60–100%) ^a^	extrusion/1000 °C	Italy	[41]
		metamorphic eluvium R (0–40%) ^a^			
		Granitoid eluvium (0–20%) ^a^			
		Granitoid tailings (0–20%) ^a^			
		Albitite tailings (0–20%) ^a^			
Suarez-Marcias et al., 2020	bricks	lead mine tailing (0–100%)	pressing 50 ± 1 MPa/950 ± 5 °C	Spain	[48]
		clay (100–0%)			
Loutou et al., 2019	bricks	red clay (100%)	molded <6 MPa/900 °C, 1000 °C, and 1100 °C	Morocco	[49]
Mendes et al., 2019	bricks	iron ore tailing (0–40%) ^b^	pressing/extrusion 40–70 MPa/850 °C, 950 °C, and 1050 °C	Brazil	[50]
		grey clay (30–70%) ^b^			
		yellow clay (30–70%) ^b^			
Li et al., 2019	porous bricks	100% iron tailing ^b^	foam-gel casting/ 1070–1120 °C	China	[51]
Ettoumi et al., 2021	brick	100% phosphate sludge	pressing 6 MPa/900 °C, 1000 °C, and 1100 °C	Tunisia	[52]
Bayoussef et al., 2021	bricks	red clay (70–100%)	pressing/1100 °C	Morocco	[53]
		fly ash (0–30%)			
Yang et al., 2014	bricks	Low silicon iron ore tailing (80–100%) ^a^	pressing 20 MPa/900 °C, 950 °C, and 1000 °C	China	[54]
		fly ash (0–20%) ^a^			
Vilela et al., 2020	soil-cement bricks	soil partial substituted with iron ore tailings 0–40%	pressing/curing 20 ± 2 °C (28 days)	Brazil	[55]
		Portland cement (10%)			
		ratio soil: cement (9:1)			
Weishi et al., 2018	brick	low-silicon iron ore tailings (75%)	molding pressure 50 MPa/curing 30–60 °C	China	[56]
		curing agent (fly ash, lime, gypsum) (25%)			
		stearic acid emulsion			
da Silva et al., 2014	red ceramic	iron tailings (0–5%)^b^	pressed 20 MPa/950 °C	Brazil	[57]
		clay (95–100%)^b^			
Luo et al., 2020	sintered brick	iron ore tailings (54%)^b^	pressing 20 MPa/950–1100 °C	China	[58]
		shale (10%) ^b^			
		coal gangue powder (30%) ^b^			
		sewage sludge (0–12%) ^b^			
Wang et al., 2019	brick	Iron tailings (40–70%) ^a^	Pressing 20 MPa/1000, 1050, 1100, 1150, or 1200 °C)	China	[59]
		Fly ash (20–50%) ^a^			
		Kaolin (10%) ^a^			
Chen et al., 2011	brick	hematite tailings (77–100%),	pressing 20–25 MPa/850, 900, 950, 980, 1000, 1030, and 1050 °C	China	[60]
		fly ash (0–8%) ^a^			
		clay (0–15%)			
Yonggang et al., 2011	bricks	fine gold tailings (60–100%) ^b^	pressed 5–20 MPa/900–1050 °C	NA	[61]
		medium gold tailings (10–30%) ^b^			
		clays (10–40%) ^b^			
Wei et al., 2021	sintered bricks	gold mine tailing (60–100%)	pressing/900–1050 °C	China	[62]
		+clay (0–40%)			

(wt %) ^a^ (mass %) ^b^.

**Table 3:** Mining waste used for AAC product.

Authors	Materials	Composition	Origin of Waste	Ref.
Huang et al., 2012	AAC	copper tailings (30%) ^a^	China	[75]
		blast furnace slag (35%) ^a^		
		quartz sand (20%) ^a^		
		cement clinker (10%) ^a^		
		gypsum (5%) ^a^		
Fang et al., 2011	Autoclaved sand-lime brick	copper tailing (0–88%) ^b^	China	[76]
		sand powder (0–15%) ^b^		
		river sand (0–88%) ^b^		
		lime (6.7–13.3%) ^b^		
Zhao, Y. 2012	Autoclaved bricks	*Optimum mixture:* hematite tailings:lime:sand ratio (70:15:15) ^b^	China	[77]
Ma et al. 2016	AAC blocks	iron tailings (0–68%) ^b^	China	[78]
		cement (8%) ^b^		
		quicklime (19–27%) ^b^		
		silicon sand (0–68%) ^b^		
		gypsum (3%) ^b^		
		Al powder (0.14%) ^b^		
Liang et al., 2019	AAC	iron tailing (30–55%) ^b^	China	[79]
		silica sand (5–30%) ^b^		
		lime (20–30%) ^b^		
		ordinary Portland cement (5–15%) ^b^		
		flue gas desulfurization gypsum (5%) ^b^		
Cai et al., 2016	AAC blocks	iron tailings (0–68%) ^b^	China	[80]
		cement (8%) ^b^		
		quicklime (21%) ^b^		
		silicon sand (0–68%) ^b^		
		gypsum (3%) ^b^		
		al powder (0.14%) ^b^		
Zhao et al., 2009	autoclaved brick	low-silicon tailings (83%) ^b^ alkali-activated slag/fly ash	China	[82]
Wang et al., 2016	AAC	coal gangue (1–40%) ^b^	China	[83]
		iron ore tailing (20–59%) ^b^		
		lime (25%) ^b^		
		cement (10%) ^b^		
		gypsum (5%) ^b^		
		Al powder (0.06%) ^b^		

(wt %) ^a^ (mass %) ^b^.

**Table 4:** Types of mining waste used for geopolymer.

Authors	Materials	Composition	Technology/ Curing Temperature	Origin of Waste	Ref.
Ahmari, S. and Zhang, L., 2012	geopolymer bricks	copper mine tailings NaOH solution (10–15 M)	forming pressure (0–35 MPa)/ 60 to 120 °C	Arizona	[95]
Ahmari S. and Zhang, L., 2013	geopolymer bricks	copper mine tailings, sodium hydroxide NaOH (15 M)	forming pressure (0–35 MPa)/ 60 to 120 °C	Arizona	[92]
Beulah et al., 2021	geopolymer bricks	iron ore tailings (50–90%);	NA	India	[93]
		GGBS (10–50%)			
		and red mud (50–90%)			
		GGBS (10–50%)			
		NaOH (8 M)			
Zhang et al. 2021	geopolymer	gold mine tailing NaOH solutions (4–12 M)	molding/75 °C	Peru	[94]

**Table 5:** Strengths–Weaknesses–Opportunities–Threats (SWOT) analysis.

Strengths	Weaknesses
**S1. Technical strengths:** ✓ base of raw materials with relatively long depletion periods, consisting of qualitatively important resources with diverse chemical compositions; ✓ chemical and mineralogical characteristics close to those of raw materials, which allows the substitution of conventional raw materials in diversified percentages depending on the technical characteristics targeted; ✓ the values of the mechanical resistances on laboratory scale are good, being dependent on the content of the mining waste from the sample mass; ✓ the physical-mechanical characteristics of samples with the addition of mining waste often exceed the limit values imposed by the design codes; ✓ samples with low content of mining waste or those burned at higher temperatures have a low content of heavy metals, thus classifying the finished products in the category of inert materials; ✓ mining tailings materials do not require grinding and screening, which can lead to increased productivity and reduced production costs. **S2. Environmental strengths:** ✓ can substitute total or partially the virgin raw material contributing to the preservation of natural resources; ✓ increasing the efficiency in the manufacturing process of the final products by consuming less natural and material resources; ✓ reduce the water, air, and land pollution, but also the disposal cost. **S3. Social strengths:** ✓ new job opportunity in the recycling supply chains; ✓ low cost of the product will increase material affordability; ✓ there is a higher and professional education infrastructure capable of preparing qualified personnel for the activity of reusing mining waste in construction. **S4. Economic strengths:** ✓ may increase the business competitivity due to product attractivity and low cost, especially in areas with scarce natural resources; ✓ emergence of potential suppliers, demanders, and end-users; **S5. Diversity in resources harnessing:** ✓ decreases the dependence on the exploitation of natural resources (clay, limestone, sand); ✓ harnessing waste as it is possible to produce new products from waste resources; ✓ harnessing diversity for global business performance; ✓ they can be an alternative to conventional materials with a higher price [106].	**W1. Technical weaknesses:** ✓ the coarse mining waste requires mechanical treatment (grinding, drying, collecting) in the preliminary stage, which can increase the energy consumption and CO2 emissions; ✓ high content of toxic substances and hazardous elements, which limit their uses in a high percent as conventional material substitution; ✓ lower characteristics compared with conventional raw materials; ✓ requires development of the existing technology, which can increase the investment cost; ✓ studies must be extended from laboratory to large-scale products to validate their technical properties; ✓ the current technical endowment does not ensure economic efficiency; ✓ inadequate access to transport infrastructure and utilities, cantonment of productive capacities in isolated, mono-industrial areas; ✓ lack of specialized post-extraction processing industries. **W2. Introduction risks:** ✓ high content of toxic substances or hazardous elements may endanger the lives of employees in brick factories; ✓ difficult operating conditions. **W3. Lack of support from the government:** ✓ lack of national regulation for secondary materials; ✓ lack of financial support for technological investments; ✓ non-existence at the national level of up-to-date and transparent statistical databases, referring to: deposits in operation, concessioned or potentially concessionable, volumes and quantities of reserves exploited annually and, implicitly, of resulting waste, economic agents carrying out activities of extraction of useful mineral substances, and waste storage; ✓ weak interest of local authorities, due to the absence of legislative provisions through which part of the fees or royalties paid by operating license holders should be directed to environmental funds, programs to restore affected areas, reuse of waste and its reintegration in the value chain. **W4. Environmental weaknesses:** ✓ treatment process requires energy consumption and CO_2_ emissions, especially for coarse waste; ✓ may contain high or medium percent of toxic substances and hazardous elements. **W5. Economic weaknesses:** ✓ the treatment process especially of coarser waste may increase the production cost; ✓ require financial supports to innovate the supply chain.
**Opportunities**	**Threats**
**O1. Technical opportunities:** ✓ the existence of a natural competitive advantage in markets in neighboring countries that do not have mineral waste reserves and the expansion of the market with the accession of new member states to the EU; ✓ the perspective of implementing new technologies for reuse of mining waste. **O2. Environmental opportunities:** ✓ recycling of mining waste to support the transition from a linear to circular economy; ✓ reuse of waste resulting in the production stage of masonry units or their recycling in the construction and demolition stages in the production of new construction materials is a viable solution to ensure the circularity of the materials; ✓ the waste resulting in the construction and demolition stages can also be reused in the construction of masonry units in the case of new buildings if they meet the quality criteria, but also in other complementary areas [107]. **O3. Social opportunities:** ✓ the existence of a potential for capitalization of some activities related to the exploitation of mining waste; ✓ collaboration opportunities between academic and research institutes or/and economic operators to contribute and to transfer the know-how to the last one [108]; ✓ creation of new jobs, especially in disadvantaged areas due to the decrease/closure of mining operations. **O4. Economic opportunities:** ✓ increased cooperation with mining industry to contribute to waste circularity and to ensure industrial symbiosis; ✓ the “green” transition is a major opportunity by creating markets for clean technologies and products, as well as creating new value chains in the construction material sectors; ✓ implementation of digital, intelligent technologies that can ensure the transition to the circular economy can effectively contribute to the sustainable development of enterprises to increase competitiveness, create jobs, and reduce the impact on the environment.	**T1. Technical threats:** ✓ content of useful substance; ✓ the existence of other types of waste possible to be used as secondary materials in construction that can lead to the development of products with technical characteristics similar or even superior to those obtained on the basis of mining waste; ✓ insufficient study of the products at the producer level, on a real scale; ✓ the need to develop pilot factories to validate the results obtained at the laboratory level; ✓ the aggregates resulting from the recycled materials have an increased porosity, so that the maximum allowed percentage is limited, so as not to affect the technical qualities of the final product. **T2. Environmental threats:** ✓ adopting mandatory reduction policies. **T3. Social threats:** ✓ increasing the health risks for the labor force involved; ✓ lack of specialized labor force; ✓ immaturity of the legislative framework; ✓ job creation in the areas of mining involved in waste recycling will lead to income security and increased living standards. **T4. Economic threats:** ✓ increasing the costs of transporting mining waste from the storage area to the factory; ✓ the need to invest in adapting the technological flow to the current requirements; ✓ the existence of other waste (organic or inorganic) generated as a result of local industrial activities can reduce the attractiveness of mining waste; ✓ low costs for the exploitation of conventional natural resources (clay, limestone, sand); ✓ sufficient fiscal instruments to support investment programs in the recycling of mining waste are not developed.

## Data Availability

Not applicable.

## References

[1] IEA (2019). Global Status Report for Buildings and Construction. https://www.iea.org/reports/global-status-report-for-buildings-and-construction-2019.

[2] Paris Agreement UN Framework Convention on Climate Change–EUR-Lex. https://eur-lex.europa.eu/content/paris-agreement/paris-agreement.html.

[3] Communication from the Commission to the European Parliament, The European Council, The Council, The European Economic and Social Committee and the Committee of the Regions The European Green Deal COM/2019/640 Final. https://eur-lex.europa.eu/legal-content/EN/TXT/HTML/?uri=CELEX:52019DC0640&from=EN.

[4] (2012). Ellen MacArthur Foundation towards the Circular Economy: Economic and Business Rationale for an Accelerated Transition. http://circularfoundation.org/sites/default/files/.

[5] Communication from the Commission to the European Parliament, The Council, The European Economic And Social Committee and the Committee of the Regions a New Circular Economy Action Plan for a Cleaner and More Competitive Europe COM/2020/98 Final. https://eur-lex.europa.eu/legal-content/EN/TXT/HTML/uri=CELEX:52020DC0098&from=EN.

[6] Norouzi M., Chàfer M., Cabeza L.F., Jiménez L., Boer D. (2021). Circular economy in the building and construction sector: A scientific evolution analysis. J. Build. Eng..

[7] Velenturf A.P.M., Archer S.A., Gomes H.I., Christgen B., Lag-Brotons A.J., Purnell P. (2019). Circular economy and the matter of integrated resources. Sci. Total Environ..

[8] Smol M., Kulczycka J., Henclik A., Gorazda K., Wzorek Z. (2015). The possible use of sewage sludge ash (SSA) in the construction industry as a way towards a circular economy. J. Clean. Prod..

[9] Atta I., Bakhoum E.S., Marzouk M.M. (2021). Digitizing material passport for sustainable construction projects using BIM. J. Build. Eng..

[10] Communication from the Commission to the European Parliament, The Council, The European Economic and Social Committee and the Committee of the Regions Towards a Circular Economy: A Zero Waste Program for Europe/COM/2014/398 Final. https://www.oecd.org/env/outreach/EC-Circular-econonomy.pdf.

[11] (2019). Circle Economy the Circularity Gap Report: Closing the Circularity Gap in a 9% World. https://www.circulareconomyclub.com/listings/the-circularity-gap-report-our-world-is-only-9-circular/.

[12] European Commission The Raw Materials Initiative—Meeting Our Critical Needs for Growth and Jobs in Europe. https://eur-lex.europa.eu/legal-content/EN/TXT/?uri=CELEX:52008DC0699.

[13] http://www.namr.ro.

[14] Marinescu M., Kriz A., Tiess G. (2013). The necessity to elaborate minerals policies exemplified by Romania. Resour. Policy.

[15] The Ministry of Economy (2017). Strategia Minieră a României 2017–2035 (Romania’s Mining Strategy 2017–2035). http://www.economie.gov.ro/images/resurse-minerale/STRATEGIE%20MINIERA%20draft%20final%2024%20OCT%202016.pdf.

[16] Eurostat Waste Generation by Economic Activity and Households. https://ec.europa.eu/eurostat/statistics-explained/index.php?title=Waste_statistics.

[17] National Agency for Environmental Protection (2019). Raport Privind Starea Mediului in Romania 2019 (Annual Report on the State of the Environment in Romania for 2019). http://www.anpm.ro/ro/raport-de-mediu.

[18] (1975). Council Directive 75/442/EEC of 15 July 1975 on Waste Management. https://eur-lex.europa.eu/eli/dir/1975/442.

[19] (2014). European Commission Commission Decision of 18 December 2014 amending Decision 2000/532/EC on the list of waste pursuant to Directive 2008/98/EC of the European Parliament and of the Council. Off. J. Eur. Union.

[20] Veiga Simão F., Chambart H., Vandemeulebroeke L., Cappuyns V. (2021). Incorporation of sulphidic mining waste material in ceramic roof tiles and blocks. J. Geochem. Explor..

[21] (2013). Seismic Design Code—Part I—Provisions for the Design of Buildings. Indicative P100-1 (In Romanian) Elaborated by UTCB, Endorsed by MDRAP.

[22] (2013). Cod de Proiectare Pentru Structuri Din Zidărie, Indicativ CR6–2013 (Design Code for Masonry Structures, Indicative CR6–2013). https://www.mdlpa.ro/userfiles/consultari_publice/24_04_13/act_16.pdf.

[23] BDA Brick Development Association. https://brick.org.uk/.

[24] Ceramie-Unie Ceramic Roadmap, Paving the Way to 2050. http://cerameunie.eu/topics/cerame-unie-sectors/cerame-unie/ceramic-industry-roadmap-paving-the-way-to-2050/.

[25] Murmu A.L., Patel A. (2018). Towards sustainable bricks production: An overview. Constr. Build. Mater..

[26] Morales M.P., Juárez M.C., López-Ochoa L.M., Doménech J. (2011). Study of the geometry of a voided clay brick using rectangular perforations to optimize its thermal properties. Appl. Therm. Eng..

[27] Morales M.P., Juárez M.C., Muñoz P., Gómez J.A. (2011). Study of the geometry of a voided clay brick using non-rectangular perforations to optimise its thermal properties. Energy Build..

[28] Sutcu M., del Coz Díaz J.J., Álvarez Rabanal F.P., Gencel O., Akkurt S. (2014). Thermal performance optimization of hollow clay bricks made up of paper waste. Energy Build..

[29] Morales M.P., Juárez M.C., López-Ochoa L.M., Muñoz P. (2012). Influence of tongue and groove system on the thermal properties of large-format voided clay bricks for single-leaf walls. Constr. Build. Mater..

[30] Pavlík Z., Jerman M., Fořt J., Černý R. (2015). Monitoring Thermal Performance of Hollow Bricks with Different Cavity Fillers in Difference Climate Conditions. Int. J. Thermophys..

[31] Kočí J., Maděra J., Jerman M., Černý R. (2015). Computational assessment of thermal performance of contemporary ceramic blocks with complex internal geometry in building envelopes. Energy Build..

[32] Li J., Meng X., Gao Y., Mao W., Luo T., Zhang L. (2018). Effect of the insulation materials filling on the thermal performance of sintered hollow bricks. Case Stud. Therm. Eng..

[33] Zukowski M., Haese G. (2010). Experimental and numerical investigation of a hollow brick filled with perlite insulation. Energy Build..

[34] Lai M.K., Maged A.S.B. (2017). Study of Sawdust and Expanded Polystyrene as Cavity Filler Material on the Effect of Thermal Conductivity in Perforated Clay Brick. Mater. Sci. Forum.

[35] Salleh S.Z., Awang Kechik A., Yusoff A.H., Taib M.A.A., Mohamad Nor M., Mohamad M., Tan T.G., Ali A., Masri M.N., Mohamed J.J. (2021). Recycling food, agricultural, and industrial wastes as pore-forming agents for sustainable porous ceramic production: A review. J. Clean. Prod..

[36] Heniegal A.M., Ramadan M.A., Naguib A., Agwa I.S. (2020). Study on properties of clay brick incorporating sludge of water treatment plant and agriculture waste. Case Stud. Constr. Mater..

[37] Kazmi S.M.S., Munir M.J., Patnaikuni I., Wu Y.F., Fawad U. (2018). Thermal performance enhancement of eco-friendly bricks incorporating agro-wastes. Energy Build..

[38] Muñoz P., Mendívil M.A., Letelier V., Morales M.P. (2019). Thermal and mechanical properties of fired clay bricks made by using grapevine shoots as pore forming agent. Influence of particle size and percentage of replacement. Constr. Build. Mater..

[39] Anjum F., Naz M.Y., Ghaffar A., Shukrullah S., AbdEl-Salam N.M., Ibrahim K.A. (2020). Study of thermal and mechanical traits of organic waste incorporated fired clay porous material. Phys. B Condens. Matter.

[40] Demir I., Baspinar M.S., Orhan M. (2005). Utilization of kraft pulp production residues in clay brick production. Build. Environ..

[41] Marrocchino E., Zanelli C., Guarini G., Dondi M. (2021). Recycling mining and construction wastes as temper in clay bricks. Appl. Clay Sci..

[42] Rehman M.U., Ahmad M., Rashid K. (2020). Influence of fluxing oxides from waste on the production and physico-mechanical properties of fired clay brick: A review. J. Build. Eng..

[43] dos Reis G.S., Cazacliu B.G., Cothenet A., Poullain P., Wilhelm M., Sampaio C.H., Lima E.C., Ambros W., Torrenti J.M. (2020). Fabrication, microstructure, and properties of fired clay bricks using construction and demolition waste sludge as the main additive. J. Clean. Prod..

[44] Cobîrzan N., Balog A.-A., Thalmaier G., Nasui M., Munteanu C., Babota F. (2020). Microscopical and Macroscopical Analysis of Recovered Bricks for Assessing Their Reusability in Masonry Buildings. Procedia Manuf..

[45] Saboya F., Xavier G.C., Alexandre J. (2007). The use of the powder marble by-product to enhance the properties of brick ceramic. Constr. Build. Mater..

[46] Cobo-Ceacero C.J., Cotes-Palomino M.T., Martínez-García C., Moreno-Maroto J.M., Uceda-Rodríguez M. (2019). Use of marble sludge waste in the manufacture of eco-friendly materials: Applying the principles of the Circular Economy. Environ. Sci. Pollut. Res..

[47] Menezes R.R., Ferreira H.S., Neves G.A., de L. Lira H., Ferreira H.C. (2005). Use of granite sawing wastes in the production of ceramic bricks and tiles. J. Eur. Ceram. Soc..

[48] Suárez-Macías J., Terrones-Saeta J.M., Iglesias-Godino F.J., Corpas-Iglesias F.A. (2020). Retention of Contaminants Elements from Tailings from Lead Mine Washing Plants in Ceramics for Bricks. Minerals.

[49] Loutou M., Taha Y., Benzaazoua M., Daafi Y., Hakkou R. (2019). Valorization of clay by-product from Moroccan Phosphate Mines for the Production of Fired Bricks. J. Clean. Prod..

[50] Mendes B.C., Pedroti L.G., Fontes M.P.F., Ribeiro J.C.L., Vieira C.M.F., Pacheco A.A., de Azevedo A.R.G. (2019). Technical and environmental assessment of the incorporation of iron ore tailings in construction clay bricks. Constr. Build. Mater..

[51] Li R., Zhou Y., Li C., Li S., Huang Z. (2019). Recycling of industrial waste iron tailings in porous bricks with low thermal conductivity. Constr. Build. Mater..

[52] Ettoumi M., Jouini M., Neculita C.M., Bouhlel S., Coudert L., Taha Y., Benzaazoua M. (2021). Characterization of phosphate processing sludge from Tunisian mining basin and its potential valorization in fired bricks making. J. Clean. Prod..

[53] Bayoussef A., Oubani M., Loutou M., Taha Y., Benzaazoua M., Manoun B., Hakkou R. (2021). Manufacturing of high-performance ceramics using clays by-product from phosphate mines. Mater. Today Proc..

[54] Yang C., Cui C., Qin J., Cui X. (2014). Characteristics of the fired bricks with low-silicon iron tailings. Constr. Build. Mater..

[55] Vilela A.P., Eugênio T.M.C., de Oliveira F.F., Mendes J.F., Ribeiro A.G.C., de S. (2020). Brandão Vaz, L.E.V.; Mendes, R.F. Technological properties of soil-cement bricks produced with iron ore mining waste. Constr. Build. Mater..

[56] Weishi L., Guoyuan L., Ya X., Qifei H. (2018). The properties and formation mechanisms of eco-friendly brick building materials fabricated from low-silicon iron ore tailings. J. Clean. Prod..

[57] da Silva F.L., Araújo F.G.S., Teixeira M.P., Gomes R.C., von Krüger F.L. (2014). Study of the recovery and recycling of tailings from the concentration of iron ore for the production of ceramic. Ceram. Int..

[58] Luo L., Li K., Weng F., Liu C., Yang S. (2020). Preparation, characteristics and mechanisms of the composite sintered bricks produced from shale, sewage sludge, coal gangue powder and iron ore tailings. Constr. Build. Mater..

[59] Wang G., Ning X., Lu X., Lai X., Cai H., Liu Y., Zhang T. (2019). Effect of sintering temperature on mineral composition and heavy metals mobility in tailings bricks. Waste Manag..

[60] Chen Y., Zhang Y., Chen T., Zhao Y., Bao S. (2011). Preparation of eco-friendly construction bricks from hematite tailings. Constr. Build. Mater..

[61] Yonggang Y., Shenhong Z., Qiuyi L., Benju Y., Yu C. Research on Making Fired Bricks with Gold Tailings. Proceedings of the 2011 International Conference on Computer Distributed Control and Intelligent Environmental Monitoring.

[62] Wei Z., Zhao J., Wang W., Yang Y., Zhuang S., Lu T., Hou Z. (2021). Utilizing gold mine tailings to produce sintered bricks. Constr. Build. Mater..

[63] Zhu M., Wang H., Liu L., Ji R., Wang X. (2017). Preparation and characterization of permeable bricks from gangue and tailings. Constr. Build. Mater..

[64] Kalpana M., Mohith S. (2020). Study on autoclaved aerated concrete: Review. Mater. Today Proc..

[65] Li X.G., Liu Z.L., Lv Y., Cai L.X., Jiang D.B., Jiang W.G., Jian S. (2018). Utilization of municipal solid waste incineration bottom ash in autoclaved aerated concrete. Constr. Build. Mater..

[66] Qu X., Zhao X. (2017). Previous and present investigations on the components, microstructure and main properties of autoclaved aerated concrete—A review. Constr. Build. Mater..

[67] Song Y., Li B., Yang E.H., Liu Y., Ding T. (2015). Feasibility study on utilization of municipal solid waste incineration bottom ash as aerating agent for the production of autoclaved aerated concrete. Cem. Concr. Compos..

[68] Drochytka R., Zach J., Korjenic A., Hroudová J. (2013). Improving the energy efficiency in buildings while reducing the waste using autoclaved aerated concrete made from power industry waste. Energy Build..

[69] Kurama H., Topçu I.B., Karakurt C. (2009). Properties of the autoclaved aerated concrete produced from coal bottom ash. J. Mater. Process. Technol..

[70] Serhat Baspinar M., Demir I., Kahraman E., Gorhan G. (2014). Utilization potential of fly ash together with silica fume in autoclaved aerated concrete production. KSCE J. Civ. Eng..

[71] El-Didamony H., Amer A.A., Mohammed M.S., El-Hakim M.A. (2019). Fabrication and properties of autoclaved aerated concrete containing agriculture and industrial solid wastes. J. Build. Eng..

[72] Różycka A., Pichór W. (2016). Effect of perlite waste addition on the properties of autoclaved aerated concrete. Constr. Build. Mater..

[73] Kunchariyakun K., Asavapisit S., Sombatsompop K. (2015). Properties of autoclaved aerated concrete incorporating rice husk ash as partial replacement for fine aggregate. Cem. Concr. Compos..

[74] Peng Y., Liu Y., Zhan B., Xu G. (2021). Preparation of autoclaved aerated concrete by using graphite tailings as an alternative silica source. Constr. Build. Mater..

[75] Huang X.Y., Ni W., Cui W.H., Wang Z.J., Zhu L.P. (2012). Preparation of autoclaved aerated concrete using copper tailings and blast furnace slag. Constr. Build. Mater..

[76] Fang Y., Gu Y., Kang Q., Wen Q., Dai P. (2011). Utilization of copper tailing for autoclaved sand–lime brick. Constr. Build. Mater..

[77] Zhao Y., Zhang Y., Chen T., Chen Y., Bao S. (2012). Preparation of high strength autoclaved bricks from hematite tailings. Constr. Build. Mater..

[78] Ma B., Cai L.-X., Li X., Jian S. (2016). Utilization of iron tailings as substitute in autoclaved aerated concrete: Physico-mechanical and microstructure of hydration products. J. Clean. Prod..

[79] Liang X., Wang C., Zhan J., Cui X., Ren Z. (2019). Study on preparation of eco-friendly autoclaved aerated concrete from low silicon and high iron ore tailings. J. New Mater. Electrochem. Syst..

[80] Cai L., Ma B., Li X., Lv Y., Liu Z., Jian S. (2016). Mechanical and hydration characteristics of autoclaved aerated concrete (AAC) containing iron-tailings: Effect of content and fineness. Constr. Build. Mater..

[81] Zhang S., Xue X., Liu X., Duan P., Yang H., Jiang T., Wang D., Liu R. (2006). Current situation and comprehensive utilization of iron ore tailing resources. J. Min. Sci..

[82] Zhao F., Zhao J., Liu H. (2009). Autoclaved brick from low-silicon tailings. Constr. Build. Mater..

[83] Wang C.L., Ni W., Zhang S.Q., Wang S., Gai G.S., Wang W.K. (2016). Preparation and properties of autoclaved aerated concrete using coal gangue and iron ore tailings. Constr. Build. Mater..

[84] Davidovits J. (1991). Geopolymers. J. Therm. Anal..

[85] Youssef N., Lafhaj Z., Chapiseau C. (2020). Economic Analysis of Geopolymer Brick Manufacturing: A French Case Study. Sustainability.

[86] Zhao J., Tong L., Li B., Chen T., Wang C., Yang G., Zheng Y. (2021). Eco-friendly geopolymer materials: A review of performance improvement, potential application and sustainability assessment. J. Clean. Prod..

[87] Mohsen Q., Mostafa N.Y. (2010). Investigating the possibility of utilizing low kaolinitic clays in production of geopolymer bricks. Ceram.-Silik..

[88] Amin S.K., El-Sherbiny S.A., El-Magd A.A.M.A., Belal A., Abadir M.F. (2017). Fabrication of geopolymer bricks using ceramic dust waste. Constr. Build. Mater..

[89] Komnitsas K., Zaharaki D., Vlachou A., Bartzas G., Galetakis M. (2015). Effect of synthesis parameters on the quality of construction and demolition wastes (CDW) geopolymers. Adv. Powder Technol..

[90] Madani H., Ramezanianpour A.A., Shahbazinia M., Ahmadi E. (2020). Geopolymer bricks made from less active waste materials. Constr. Build. Mater..

[91] Kang X., Gan Y., Chen R., Zhang C. (2021). Sustainable eco-friendly bricks from slate tailings through geopolymerization: Synthesis and characterization analysis. Constr. Build. Mater..

[92] Ahmari S., Zhang L. (2013). Durability and leaching behavior of mine tailings-based geopolymer bricks. Constr. Build. Mater..

[93] Beulah M., Sudhir M.R., Mohan M.K., Gayathri G., Jain D. (2021). Mine Waste-Based Next Generation Bricks: A Case Study of Iron Ore Tailings, Red Mudand GGBS Utilization in Bricks. Adv. Mater. Sci. Eng..

[94] Zhang N., Hedayat A., Bolaños Sosa H.G., González Cárdenas J.J., Salas Álvarez G.E., Ascuña Rivera V.B. (2021). Damage evaluation and deformation behavior of mine tailing-based Geopolymer under uniaxial cyclic compression. Ceram. Int..

[95] Ahmari S., Zhang L. (2012). Production of eco-friendly bricks from copper mine tailings through geopolymerization. Constr. Build. Mater..

[96] Zhang N., Tang B., Liu X. (2021). Cementitious activity of iron ore tailing and its utilization in cementitious materials, bricks and concrete. Constr. Build. Mater..

[97] Cicek B., Karadagli E., Duman F. (2018). Valorisation of boron mining wastes in the production of wall and floor tiles. Constr. Build. Mater..

[98] Yun-Ming L., Cheng Yong H., Mustafa A.B.M., Hussin K. (2016). Structure and Properties of Clay-Based Geopolymer Cements: A Review. Prog. Mater. Sci..

[99] Muñoz Velasco P., Morales Ortíz M.P., Mendívil Giró M.A., Muñoz Velasco L. (2014). Fired clay bricks manufactured by adding wastes as sustainable construction material–A review. Constr. Build. Mater..

[100] Muñoz P., Letelier V., Zamora D., Morales M.P. (2020). Feasibility of using paper pulp residues into fired clay bricks. J. Clean. Prod..

[101] Görhan G., Şimşek O. (2013). Porous clay bricks manufactured with rice husks. Constr. Build. Mater..

[102] Steinmann Z.J.N., Huijbregts M.A.J., Reijnders L. (2019). How to define the quality of materials in a circular economy?. Resour. Conserv. Recycl..

[103] Elert K., Cultrone G., Navarro C.R., Pardo E.S. (2003). Durability of bricks used in the conservation of historic buildings—influence of composition and microstructure. J. Cult. Herit..

[104] García-Ten J., Orts M.J., Saburit A., Silva G. (2010). Thermal conductivity of traditional ceramics. Ceram. Int..

[105] De Bonis A., Cultrone G., Grifa C., Langella A., Leone A.P., Mercurio M., Morra V. (2017). Different shades of red: The complexity of mineralogical and physico-chemical factors influencing the colour of ceramics. Ceram. Int..

[106] Megyesi E., Brumaru M., Naghiu G.S., Felseghi R.A. Choosing the optimal type of external wall constructions for application in the field of passive houses. Proceedings of the 14th International Multidisciplinary Scientific GeoConference SGEM.

[107] Horckmans L., Nielsen P., Dierckx P., Ducastel A. (2019). Recycling of refractory bricks used in basic steelmaking: A review. Resour. Conserv. Recycl..

[108] Cretu M., Ceclan A., Czumbil L., Şteţ D., Bârgăuan B., Micu D.D. Key performance indicators (KPIs) for the evaluation of the demand response in the Technical University of Cluj-Napoca buildings. Proceedings of the 8th International Conference on Modern Power Systems (MPS).

[109] European Commission Closing the Loop–An EU Action Plan for the Circular Economy COM/2015/0614 Final. http://eur-lex.europa.eu/legal-content/EN/TXT/?uri=CELEX:52015DC0614.

[110] Communication from the Commission to the European Parliament, The European Council, The Council, The European Economic and Social Committee, The Committee of the Regions and the European Investment Bank A Clean Planet for All. A European Strategic Long-Term Vision for a Prosperous, Modern, Competitive and Climate Neutral Economy COM/2018/773. https://eur-lex.europa.eu/legal-content/EN/TXT/PDF/?uri=CELEX:52018DC0773.

[111] Zhang L. (2013). Production of bricks from waste materials–A review. Constr. Build. Mater..

[112] Huysman S., Debaveye S., Schaubroeck T., De Meester S., Ardente F., Mathieux F., Dewulf J. (2015). The recyclability benefit rate of closed-loop and open-loop systems: A case study on plastic recycling in Flanders. Resour. Conserv. Recycl..

